# Photic Desynchronization of Two Subgroups of Circadian Oscillators in a Network Model of the Suprachiasmatic Nucleus with Dispersed Coupling Strengths

**DOI:** 10.1371/journal.pone.0036900

**Published:** 2012-05-16

**Authors:** Changgui Gu, Zonghua Liu, William J. Schwartz, Premananda Indic

**Affiliations:** 1 Institute of Theoretical Physics and Department of Physics, East China Normal University, Shanghai, China; 2 Department of Neurology, University of Massachusetts Medical School, Worcester, Massachusetts, United States of America; Vanderbilt University, United States of America

## Abstract

The suprachiasmatic nucleus (SCN) is the master circadian clock in mammals and is composed of thousands of neuronal oscillators expressing different intrinsic periods. These oscillators form a coupled network with a free-running period around 24 h in constant darkness and entrainable to the external light-dark cycle (T cycle). Coupling plays an important role in setting the period of the network and its range of entrainment. Experiments in rats have shown that two subgroups of oscillators within the SCN, a ventrolateral (VL) subgroup that receives photic input and a dorsomedial (DM) subgroup that is coupled to VL, can be desynchronized under a short (22-h) T cycle, with VL entrained to the cycle and DM free-running. We use a modified Goodwin model to understand how entrainment of the subgroups to short (22-h) and long (26-h) T cycles is influenced by light intensity, the proportion of neurons that receives photic input, and coupling heterogeneity. We find that the model’s critical value for the proportion of photically-sensitive neurons is in accord with actual experimental estimates, while the model’s inclusion of dispersed coupling can account for the experimental observation that VL and DM desynchronize more readily under the 22-h than under the 26-h T cycle. Heterogeneous intercellular coupling within the SCN is likely central to the generation of complex behavioral patterns.

## Introduction

Circadian (∼24 h) rhythms in physiological and behavioral measures are universal in living things, reflecting the period of the earth’s rotation. In mammals, circadian rhythms are regulated by a master clock in the suprachiasmatic nucleus (SCN) of the hypothalamus, composed of approximately 20,000 neuronal oscillators; SCN neurons are nonidentical, express different intrinsic periods, and are coupled together to form a network with a coherent output [1]. The period of the network’s output signal is adaptable. Under constant darkness, the rhythm has a free-running period close to 24 h; whereas under an external light-dark cycle (T cycle), it is precisely entrained to a period identical to the external cycle.

The SCN network is heterogeneous [2], [3], [4]. It can be divided into distinct functional subgroups, including a ventrolateral part (VL), which receives photic input from the retina, and a dorsomedial part (DM), which is coupled to VL; both VL and DM contribute to the generation of overt circadian rhythms in physiological and behavioral measures. Peptide neurotransmitters differ between the VL and DM subdivisions, with neurons that express vasoactive intestinal polypeptide (VIP) in the VL and arginine vasopressin in the DM. Periods may vary in different regions of the SCN, with DM running faster than VL in tissue slices [5]. Gamma aminobutyric acid (GABA) neurons are present throughout the SCN and may play a role in coupling the two subdivisions [6]. It has been shown that the circadian oscillation between VL and DM can desynchronize with exposure to short T cycles [7] or after a phase shift of the light-dark cycle [6], [8], [9]; the VL appears to set the final phase of the SCN after the phase shift [6], [8], [9].

Much experimental [10], [11], [12] and theoretical [13], [14], [15] work has been motivated by a desire to understand how this heterogeneous SCN network is reliably entrained and able to generate a coherent output signal, and neuropeptidergic mechanisms appear to be necessary elements [16], [17], [18]. Modeling studies suggest that the circadian clock’s free-running period is proportional to the average intercellular coupling strength [13] and that coupling governs the clock’s range of entrainment to T cycles [15]. However, coupling strength between cells in the SCN network is unlikely to be uniform. The effects of heterogeneous coupling on network synchronization have been studied previously in multi-oscillator models. Daido considered the dispersion of coupling strengths in the Kuramoto model and studied the synchronization property of the network [19], [20]; coupling strength between two oscillators was chosen from a normal distribution. Hong and Strogatz considered a heterogeneous network with excitatory (positive) and inhibitory (negative) coupling in the Kuramoto model to understand the relative contributions of excitatory and inhibitory properties on network synchronization [21]. Our recent work (C.G. and Z.L.) has demonstrated that the dispersion of coupling strengths between SCN cellular oscillators can influence the emergent free running period of the network [22]. To our knowledge, however, there has been no work on the relationship between coupling dispersion and network entrainment.

We examine this issue in the present work, inspired by an interesting experiment performed by de la Iglesia et al. [7] in which rats were exposed to an artificially short 22-h T cycle (11 h light alternating with 11 h darkness). Individual animals expressed two separate circadian motor activity rhythms, with one rhythm entrained by the light and oscillating with a period equal to the external cycle, while the other was not entrained and expressed a period greater than 24 h. Analyses of SCN gene expression suggested that these two motor activity rhythms reflected the stable forced desynchronization of VL and DM subdivisions, respectively. Here we model how entrainment of the subdivisions is influenced by coupling dispersion, as well as by the proportion of cellular oscillators that receive photic input (i.e., that are within VL) and the light intensity.

We use the Goodwin model, a network model of coupled oscillators that has been widely used to describe the mammalian circadian clock [13], [22], [23], [24], [25], [26] (defined in Methods). An individual cellular oscillator of the Goodwin model has three variables: a clock gene mRNA, a clock protein, and a transcriptional inhibitor, all of which form a transcription-translation negative feedback loop. It is assumed that light induces the clock gene mRNA, that a neurotransmitter is increased by the clock gene mRNA, and that neurotransmitters from different neurons form a mean field that couples the neurons together. We consider that 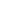 neurons receive photic input, where 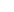 is the total number of neurons in the SCN network and 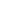 is the ratio of the number of VL neurons to the total number of SCN neurons. We take T cycles of 22 h and 26 h as examples, i.e., symmetrically distant from 24 h. We chose mean field coupling for all of the neurons in the Goodwin model. The coupling strength 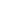 of all the 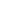 neurons satisfies a normal distribution with mean value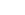 and deviation 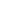.

## Results

### T-cycle Entrainment of an SCN Network without Dispersion of Coupling Strengths

**Figure 1:**
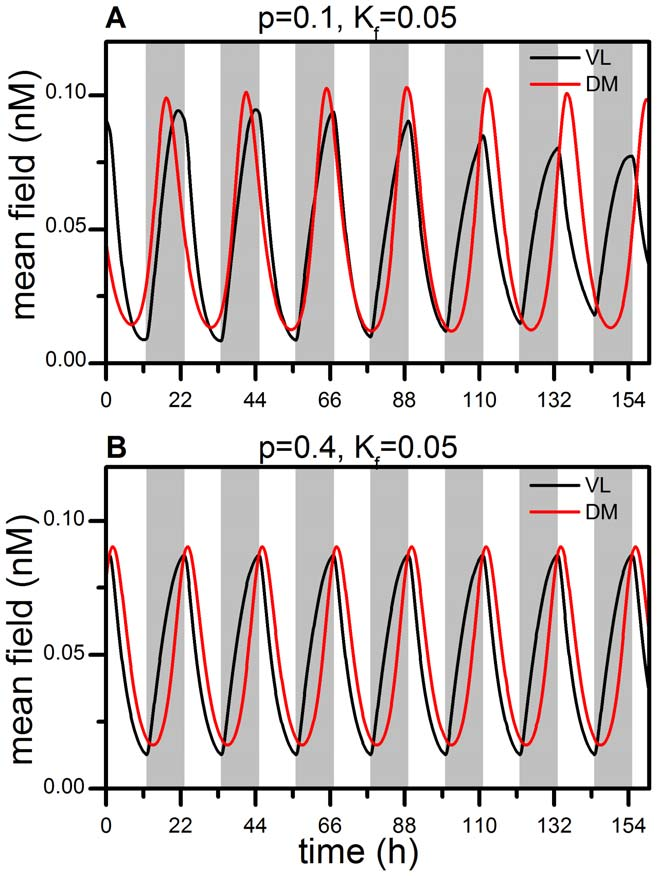
Mean field oscillations of VL and DM during a 22-h T cycle. (**A**) VL follows the T cycle, whereas DM free runs for the parameters 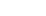 and 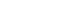. (**B**) Both VL and DM follow the T cycle for the parameters 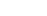 and 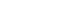. The dispersion of the coupling strengths, η, is set to zero in both (**A**) and (**B**). The grey bar indicates the dark phase, and the white bar the light phase, of the T cycle.

To determine the effects of 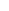 and light intensity, 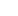, on the entrainment of VL and DM to T cycles, we have numerically simulated the Goodwin model with no dispersion of coupling strengths, i.e., 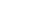. Figure 1 shows the mean field time series of VL and DM oscillations in the 22-h light-dark cycle. Similar to previous observations [13], we find that the time series show quasi-periodic behavior with low light intensity. In (A), the behavior of VL follows the 22-h cycle and sustains a stable phase relationship to it, while the behavior of DM loses its phase relationship to the cycle and runs with a period close to the intrinsic period of the network. This dissociation mimics the forced desynchronization of motor activity rhythms in rats under such a T cycle, as noted previously [7], [27]. When 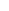 is increased, both VL and DM can be entrained, as in (B). Here the peak of the mean field time series of VL appears around the onset of darkness, whereas that of the DM is phase delayed. This change also can be implemented by 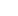. If 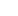 is reduced, neither VL nor DM entrain to the 22-h cycle. If 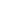 is increased, both VL and DM can be entrained, as in (B). In sum, both the number of neurons receiving light and the light intensity are important factors for entrainment of the entire SCN network to the T-cycle.

**Figure 2:**
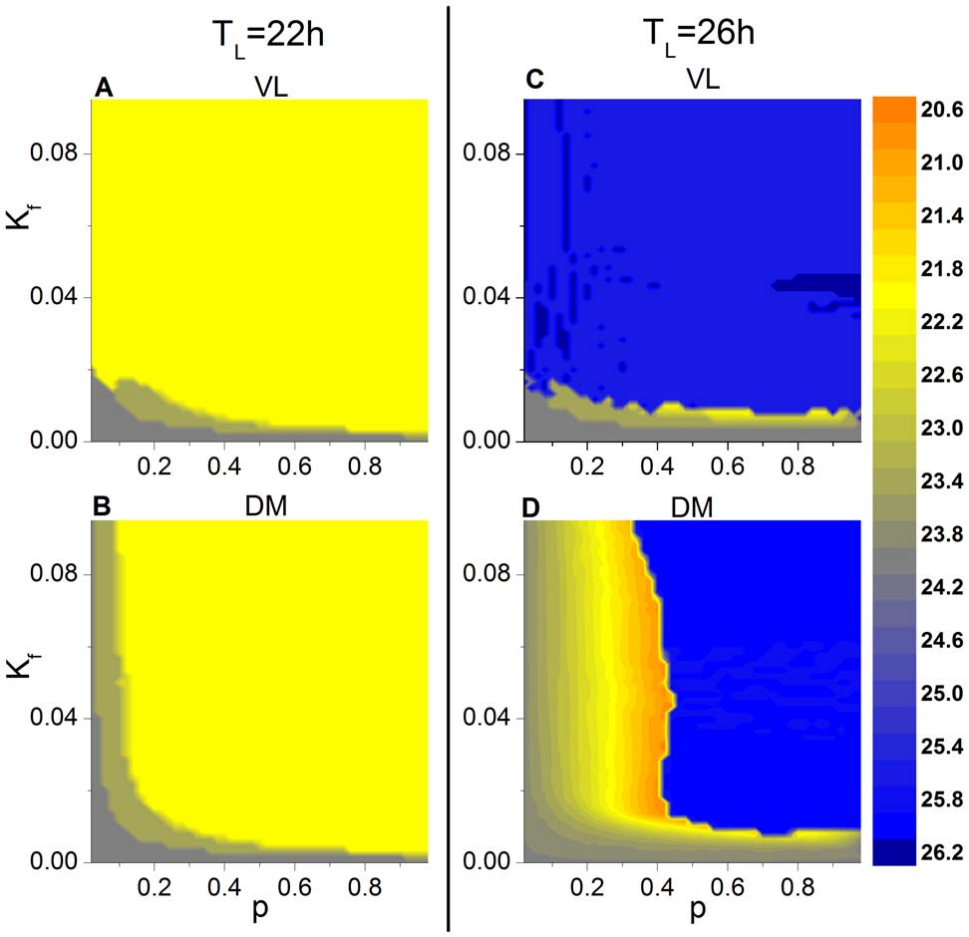
Period of the mean fields of VL and DM in the *p*−*K_f_* plane. The case for the 22-h T cycle is shown for VL (**A**) and DM (**B**), and the case for the 26-h T cycle is shown for VL (**C**) and DM (**D**). The coupling strengths are identical for all the oscillators (i.e., η = 0). Entrainment of the sub-network to the 22-h cycle is represented by the yellow region, and entrainment of the sub-network to the 26-h cycle is represented by the blue region.

To understand the influence of the parameters 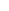 and 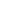 on entrainment, we have calculated the phase diagram of the period of the mean fields of VL and DM in the 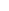-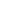 plane under short and long T cycles, i.e., of 22 h and 26 h, with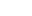 (Figure 2). (A) and (C) show that the behavior of VL follows the T cycle for all values of 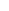, provided that the light intensity is greater than a critical value, such as 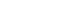. When 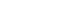, the period of VL may not be entrained to the T cycle, depending on 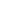; for example, in Fig. 2A, the period of VL can be 23 h or 24 h. For DM to follow the cycle, however, 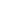 also must be larger than some threshold; that is, there must be a sufficient number of light-receiving neurons in VL in order to drive the neurons in DM. For a given level of 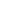, increasing 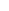 may allow the entire network to entrain to the driving T cycle. Surprisingly, when 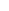 is decreased under the 26-h T cycle (D), there is a threshold of 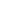 at which the period of DM suddenly jumps to a value of 20.8 h to form a locking ratio of 4∶5 with the 26-h T cycle; with further decreased 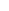, the period monotonically increases to reach a value of 24 h at 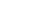.

A comparison of (B) and (D) shows that the threshold of 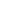 for entrainment of DM to the 26 h T cycle is greater than that for the 22 h cycle, suggesting that desynchronization between VL and DM might be more likely under long than under short T cycles. Experimentally, however, this appears not to be the case [28], [29], prompting us to consider the influence of heterogeneous coupling strengths on the behavior of the SCN network.

### T-cycle Entrainment of an SCN Network with Dispersion of Coupling Strengths

**Figure 3:**
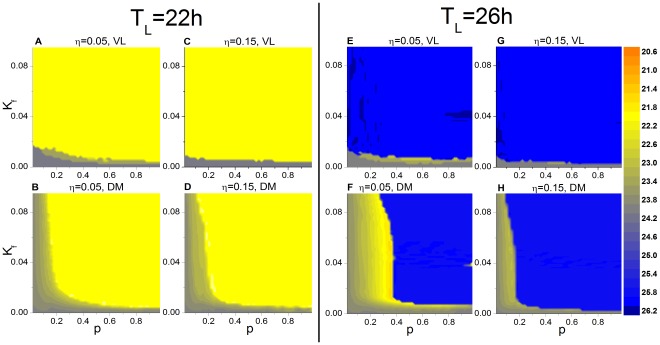
Effect of coupling dispersion on the period of the mean fields of VL and DM in the *p*−*K_f_* **plane.** The case for the 22-h T cycle is shown for VL (**A**) and DM (**B**) with 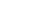 and for VL (**C**) and DM (**D**) with 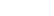. The corresponding case for the 26-h T cycle is represented in (**E**) - (**H**).

Figure 3 shows the phase diagram of the period of the mean fields of VL and DM in the 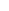-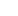 plane using two values for 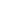. Although qualitatively similar to the diagrams in Figure 2, there are quantitative differences when network coupling strengths are dispersed. In the case of the 22-h T cycle, entrainment is only modestly affected; in contrast, in the case of the 26-h T cycle, increased coupling dispersion significantly reduces the critical value of 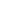 for DM entrainment, suggesting that the network can be entrained to the long T cycle with a relatively lower 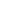.

**Figure 4:**
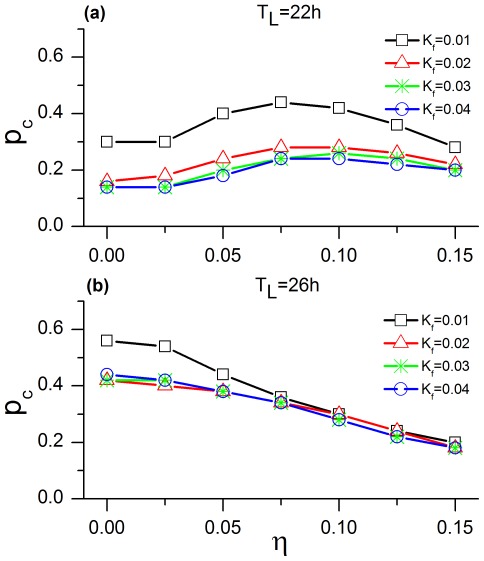
Effect of coupling dispersion on the critical *p*. Shown are the cases for the 22-h (**A**) and 26-h (**B**) T cycles.

For weak 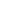 and different values of 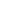, we find that the critical 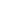 (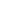) for the 22-h and 26-h T cycles reaches approximately the values of 0.28 and 0.20, respectively. Figure 4 represents the variation in 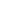 for different values of 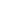 and 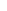. In the case of the 22-h T cycle (A), there is little variation in 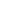 for different 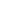, e.g., 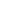 is between 0.14 and 0.24 for a 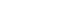. However, 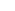 does change significantly in the case of the 26-h T-cycle (B). Thus, dispersion of coupling strengths affects entrainment in an asymmetric way, with an influence that is larger for the long than for the short T cycle.

Instead of randomly assigning coupling values to the network, we also studied the network by selectively assigning coupling values. In two separate trials, we assigned the strongest coupling values to either VL or DM. The phase diagram of the period of the mean fields of VL and DM in the 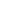
–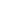
plane was similar to that previously reported.

We also simulated the network with dispersed oscillator periods, rather than dispersed coupling strengths, by selecting different values for the standard deviation of period (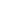) for each individual oscillator, such that the period distribution has a mean of 24 h with variability. Without dispersed coupling, we do not observe DM entrainment to the long T cycle at any 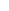 until 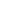 is increased to a value greater than 5 h. Since such a large non-identical intrinsic period is not realistic, the dispersion of coupling strengths is likely a crucial factor affecting the entrainment of the network to different T cycles.

**Figure 5:**
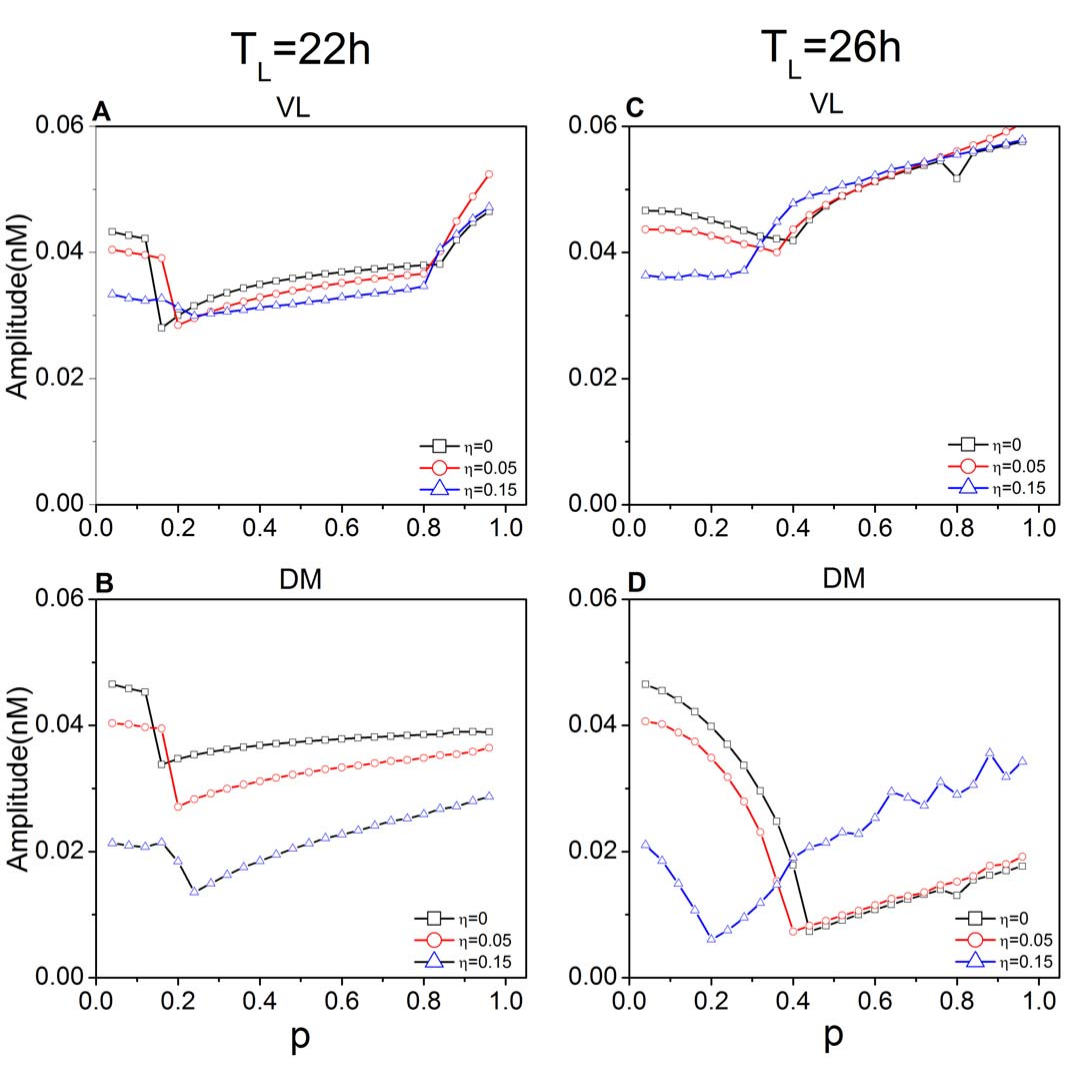
Effect of coupling dispersion on the amplitude of the mean fields of VL and DM. The case for the 22-h T cycle is shown for VL (**A**) and DM (**B**), and the case for the 26-h T cycle is shown for VL (**C**) and DM (**D**).

Importantly, dispersion of coupling influences the mean field amplitude (Figure 5). The amplitude of VL as a function of 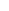 changes modestly as the dispersion 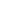 is increased in the 22-h as well as the 26-h T cycle (A and C). On the other hand, for DM in the 22-h T cycle (B), amplitude decreases as 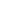 increases. In the 26-h T cycle (D), DM amplitude as a function of 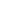 changes dramatically, with relatively diminished amplitude as 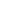 is increased; dispersion 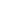 counteracts this effect. The enhancement of DM amplitude by increased 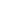 in the 26-h cycle could be due to enhanced phase synchronization of the oscillators in the network, increased amplitude of the individual oscillators, or both. To begin to distinguish among these possibilities, we studied the effect of dispersed coupling on the order parameter, a measure that represents phase synchronization of the network.

### Effect of Coupling Dispersion on the Order Parameter of the Network

Order parameter characterizes the synchronization property of a network [30], [31], and it is defined here by estimating the phases of the oscillators in VL and DM (see Methods). The order parameter will be unity if all oscillators in the network are perfectly synchronized and zero if they are completely uncorrelated. When their behavior is between these two extremes, the order parameter will be in (0, 1), i.e., representing a phase difference between VL and DM or desynchronization of individual oscillators within VL and/or DM.

**Figure 6:**
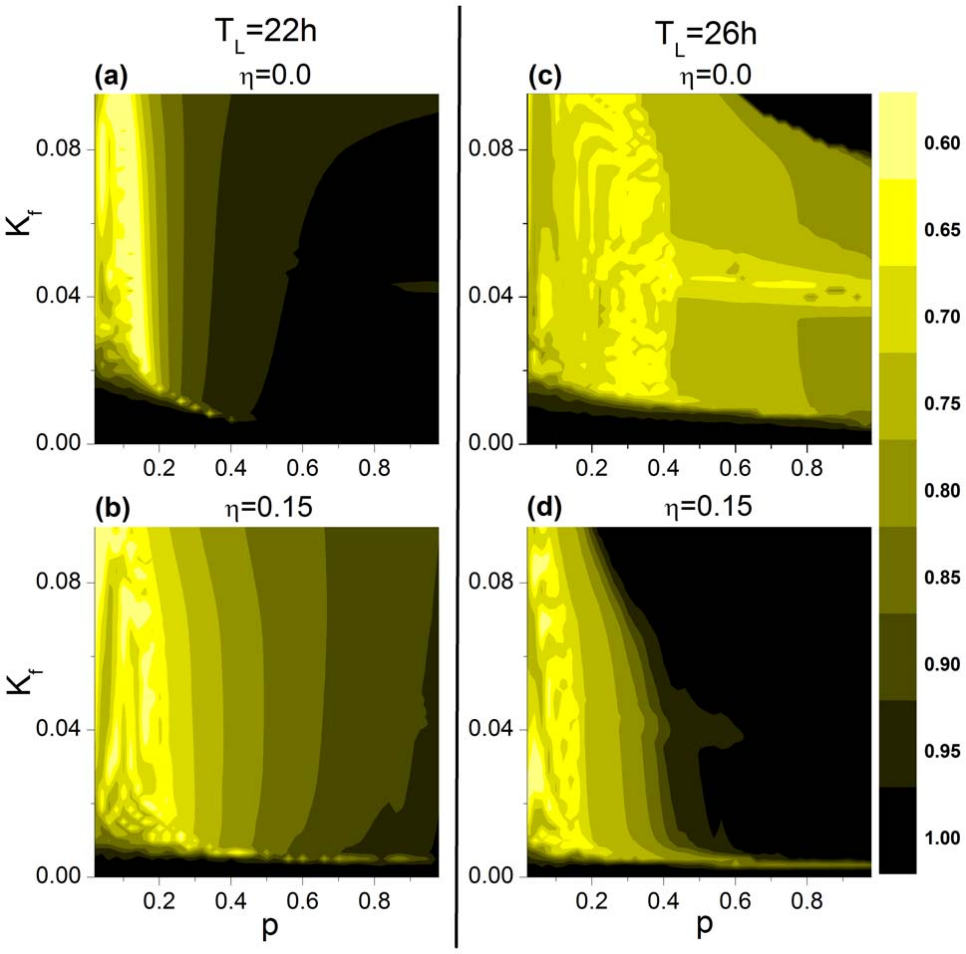
Effect of coupling dispersion on the order parameter of the network. The case for the 22-h T cycle is shown with coupling dispersion 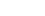 (**A**) and 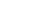 (**B**), and the case for the 26-h T cycle is shown with coupling dispersion 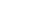 (**C**) and 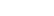 (**D**).

We have studied the influence of 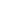 on the order parameter. Figure 6 shows the dependence of order parameter 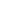 on the parameters 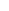 and 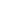 in the 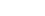 plane. To reveal the effect of coupling dispersion, we have considered two cases, one with 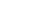 and the other with 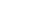. Under the 22-h T cycle, coupling dispersion reduces 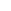 for larger 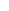 and 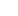 values; whereas under the 26-h T cycle, coupling dispersion enhances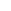. As 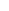 and 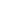 increase from (0,0), the relationship between VL and DM changes; comparison of Figure 6 with Figures 2 and 3 visualizes the regions where 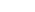, i.e., either when VL and DM express different periods or when VL and DM express the same period but with a large phase difference between them. Thus, for the 22-h cycle, although higher 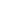 and 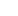 values enhance both VL and DM entrainment to the cycle, the reduction of 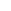 with dispersed coupling suggests that individual oscillators are not fully synchronized within the network, with a greater vulnerability to perturbations of light intensity. For the 26-h cycle, coupling dispersion synchronizes network oscillation for 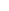
>0.6; the gradual increase of DM mean field amplitude as 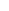 increases further (Figure 5D) is thus attributable to increased individual oscillator amplitude.

These considerations imply that VL and DM desynchronize more readily under the 22-h than under the 26-h T cycle and that dispersion of coupling strengths improves network robustness preferentially under the 26-h cycle.

### Effect of Coupling Dispersion on the Network’s Phase Response Curve to a Light Pulse

**Figure 7:**
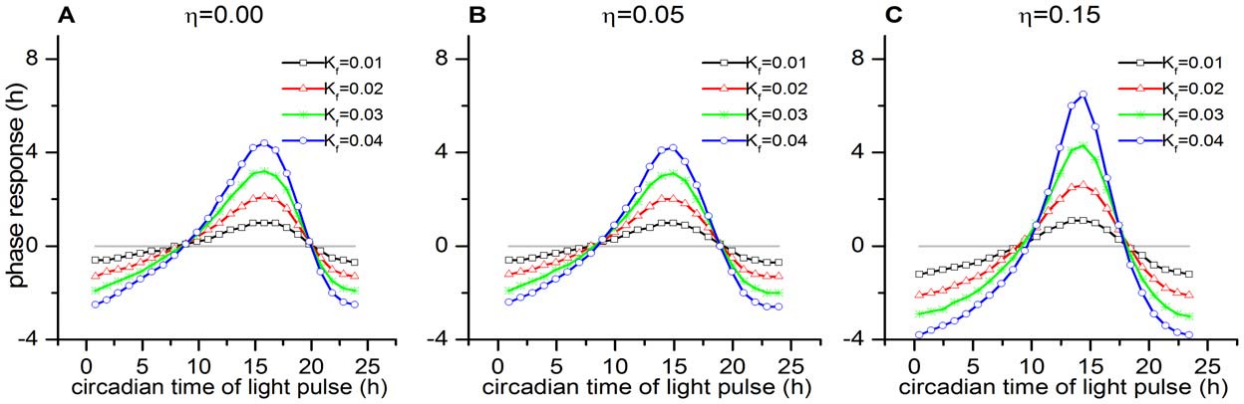
Effect of coupling dispersion on the phase response curve (PRC) of the network. Shown are PRCs with coupling dispersions 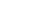 (**A**), 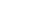 (**B**), and 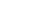 (**C**). Although the network shows relatively larger phase advances and delays with increased coupling dispersion, the area under the phase delay zones is greater than that under the advance zones. The PRCs were similar for all the values of 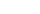.

The network’s capacity to generate phase advances or delays can be quantified as a phase-response curve (PRC), measured by plotting the phase shifts that occur in the rhythm when discrete light pulses are applied at different phase points across the circadian cycle [24], [32], [33], [34]. Figure 7 represents the family of PRC’s obtained to a 1-h light pulse of increasing intensities, showing that the phase response region (i.e., the area under the delay and advance zones) increases in magnitude with increasing 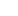. Notably, as the value of 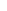 increases, the area under the delay zone increases relatively more than that under the advance zone, as calculated in Table 1, where 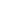 represents the ratio of the area under the delay zone to the area under the advance zone. Advances should correspond to the capacity of the network to follow a T cycle less than 24 h, while delays should correspond to its capacity to follow a T cycle greater than 24 h [35].

**Table 1:** Effect of coupling dispersion on the ratio of the area of the delay zone to the advance zone.

*η K_f_*	0.01	0.02	0.03	0.04
**0.00**	**0.69**	**0.70**	**0.71**	**0.73**
**0.05**	**0.82**	**0.85**	**0.89**	**0.92**
**0.15**	**1.80**	**1.40**	**1.40**	**1.40**

The ratio increases with increasing coupling dispersion.

## Discussion

Here we analyze the photic desynchronization of two subgroups of circadian oscillators in a network model of the suprachiasmatic nucleus. As also demonstrated in experiments with rats exposed to a short T cycle of low light intensity [7], [36], a subgroup of oscillators receiving photic input (VL) can entrain to the external cycle while the other, coupled subgroup (DM) expresses an unentrained period greater than 24 h.

Granada et al. [37] have modeled this forced desynchronization of rat activity rhythms as a single oscillator with oscillatory interactions (modulation and superposition) between the external cycle and the internal clock, while Schwartz et al. [38] have modeled entrainment to the T cycle by two coupled oscillators forced by a Zeitgeber. Casiraghi et al. [39] have used a two oscillator model to analyze a chronic jet lag paradigm that leads to forced desynchrony, and they observed an asymmetry in its behavior similar to our findings reported here. We have taken the Goodwin model and extended it to include 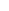, the proportion of all SCN cellular oscillators that receive photic input, and 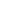, the dispersion of coupling strengths. We find, first, that network desynchronization (with an entrained VL but an unentrained DM) depends on light intensity and the value of 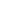. Relatively higher light intensities protect the network from desynchronization, as reported experimentally [36]. Experiments estimate that the value of 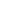 for the rodent SCN ranges from 20% to 33%, based on molecular, electrophysiological, and computational studies [40], [41]. Comparing these results to our simulations in Figure 4, we find that 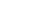 is a good parameter value to fit the experiments. At this value, there is a critical value of 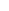 for network entrainment to short (22 h) and long (26 h) T cycles of 0.28 and 0.20, respectively, and the critical value appears fairly insensitive to 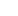. We predict that the rat SCN is likely to have this very large heterogeneity in coupling strengths, given that the critical 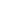 best matches the experimental estimate for the larger values of 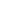.

Second, we find that the inclusion of dispersed coupling strengths affects network entrainment in a preferential manner, such that increased 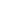 significantly reduces the critical value of 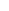 for DM entrainment to the 26-h T cycle. A consequence of this 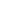 influence is that network robustness is superior under the 26-h cycle while desynchronization is favored under the 22-h cycle. In fact, such an asymmetry has been observed experimentally, with no obvious desynchronization of rat motor activity rhythms during exposure to long T cycles [28].

The basis for this asymmetry is unclear. Coupling dispersion appears to generally increase the effect of light on the system, and since the delay zone of the PRC is greater than the advance zone, a preferential effect on entrainment to the 26-h T cycle might be expected. But such an explanation does not account for the u-shaped, rather than monotonic, 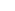 function for the 22-h T cycle. Moreover, differences in entrainment to short and long T cycles may not be a general feature of the SCN; it may not be true for other species [42], and the possible effects of locomotor activity itself on SCN network behavior (e.g., in or out of a running wheel, diurnal or nocturnal activity pattern) needs further investigation.

Our findings may provide new elements to the theory of coupled oscillators, especially with regard to chimera states in which one group of the system is synchronized and the other is desynchronized [43], [44], [45], [46], [47]; in these studies, the oscillators in the two groups are identical and the chimera states are generally induced by the initial conditions. However, in our case, the discovery that the subgroup VL may be entrained to the T cycle while the group DM remains free-running is similar to the chimera state, but this phenomenon does not depend on the initial conditions. Thus, a novel oscillator theory is needed to explain this robustness to initial conditions and should be a topic for further studies.

Heterogeneous intercellular coupling within the SCN is likely central to the generation of complex behavioral patterns. Non-uniform SCN network architecture also has been implicated in the phase-“splitting” of locomotor activity cycles seen in hamsters maintained in constant environmental light [48]. In the future, we hope to consider how topology influences entrainment, in contrast to the mean field used here.

## Methods

We represent each mammalian cell of the network as a Goodwin oscillator. The Goodwin model is a widely used mathematical model to represent the behavior of the gene regulatory network in single cellular circadian oscillators [13]. The model represents the transcription-translation behavior of the single cell by using three variables that include a clock gene mRNA (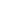), a clock protein (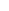), and a transcriptional inhibitor (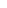).

As our network model, we consider the mean field-modified Goodwin model proposed by [13] with a global coupling strength. The modified Goodwin model with 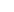 oscillators is represented as follows:
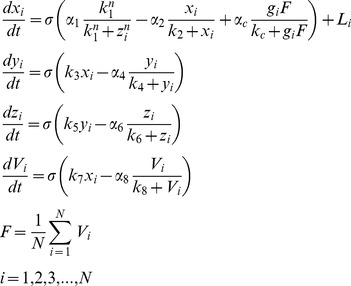
(1)Where the state variables 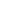, 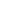, 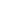 represent the concentrations, respectively, of a clock gene mRNA, a clock protein and a transcriptional inhibitor in each clock cell 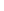. Neurotransmitter 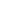 is induced by the mRNA 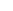. The intercellular coupling is implemented through the neurotransmitter 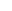 which acts as a mean field of 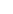, the coupling strength 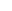 represents the sensitivity of the individual oscillator to the neurotransmitter and is required to be a positive value here, and 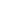 is the light term. We considered the parameters as in [13]:



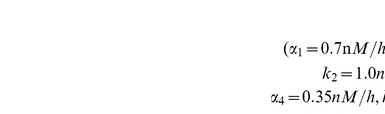



The coupling strength 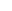 is different for different oscillators and assumes a value from a normal distribution with a mean 0.5 and a standard deviation 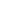. When 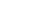, the network is heterogeneous with distribution of coupling.

In order to understand the dissociation behavior observed under a T cycle outside the range of entrainment, we modified the Goodwin model to include the fact that light acts directly on only a portion of the neurons in the network. Furthermore, the light term 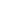 that is applied to a fraction 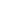 neurons with 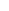 being less than one and positive, is considered to be located in the VL subdivision. Mathematically, the effect of light is represented as:
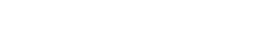
Where *T_L_* is the period of the light-dark cycle and *K_f_* is the light intensity.

As pointed out in our previous paper [22], the dispersion of coupling strengths influences the free-running period of the SCN. In order to compare the influence of different coupling dispersions on entrainment of the SCN network to T cycles, it is necessary to make the free-running period the same for different dispersions. To set the free-running period to 24 h, we multiply a rescaling factor 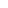 to the left hand of equation (1) except for the light term and coupling term, i.e., multiply the same 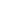 to the parameters 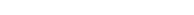 for the deviation 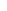. For example, 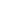 is equal to 1.26 for 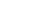, 1.22 for 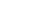, 1.16 for 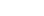, and 1.13 for 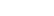.

For simplicity, we refer to the network that is comprised of *pN* neurons as VL and the network comprised of the remaining 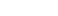 neurons as DM. To understand the behavior of the VL and DM subdivisions, we define the mean field of VL and DM respectively as
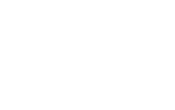



In addition, to understand the synchronization properties between VL and DM, we have estimated the phase of the individual neurons by using the Hilbert transform [49], [50]. From the estimated phase of VL and DM, we introduce an order parameter as:
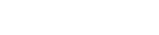
where 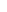 is the estimated phase from the mean field output time series of VL or DM and 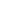 denotes average over time. The average of 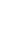 is defined as the angular frequency and the period is obtained by 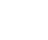. To determine entrainment of VL or DM to the 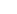, we estimated the period of VL or DM and estimated its absolute difference from 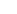. If the absolute difference in period is less than 0.25 h, the corresponding subgroup (VL or DM) is considered to be entrained. To numerically calculate the equations, we use the fourth order Runga-Kutta method with time step of 0.1 h. Initial 20000 time steps are neglected to avoid the effect of transients. The number of oscillators is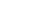, and the simulations are performed five times, with initial conditions selected randomly from a uniform distribution in the range (0–1) for 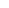, 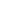, and 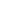. We have also calculated the case of 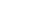 and time step of 0.01 h. Two additional simulations are performed with selective assignment of coupling in which larger values are assigned to either VL or DM without changing the intrinsic distribution of 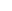.

To obtain the phase-response curve, we applied 1-h light pulses at different phases to the model, with intensity 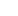 and with different values for 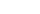. The corresponding advance or delay is detected from the output of the network. Advance corresponds to the capacity of the SCN network to follow a light-dark cycle with period less than the free running period, and delay is the capacity of the network to follow a light-dark cycle with period greater than the free running period [35].
